# A Novel Staining Protocol for Multiparameter Assessment of Cell Heterogeneity in *Phormidium* Populations (Cyanobacteria) Employing Fluorescent Dyes

**DOI:** 10.1371/journal.pone.0055283

**Published:** 2013-02-20

**Authors:** Daria Tashyreva, Josef Elster, Daniela Billi

**Affiliations:** 1 Department of Botany, Faculty of Science, University of South Bohemia, České Budějovice, Czech Republic; 2 Centre for Phycology, Institute of Botany, Academy of Sciences of the Czech Republic, Třeboň, Czech Republic; 3 Department of Biology, University of Rome “Tor Vergata”, Rome, Italy; Royal Netherlands Institute of Sea Research (NIOZ), The Netherlands

## Abstract

Bacterial populations display high heterogeneity in viability and physiological activity at the single-cell level, especially under stressful conditions. We demonstrate a novel staining protocol for multiparameter assessment of individual cells in physiologically heterogeneous populations of cyanobacteria. The protocol employs fluorescent probes, i.e., redox dye 5-cyano-2,3-ditolyl tetrazolium chloride, ‘dead cell’ nucleic acid stain SYTOX Green, and DNA-specific fluorochrome 4′,6-diamidino-2-phenylindole, combined with microscopy image analysis. Our method allows simultaneous estimates of cellular respiration activity, membrane and nucleoid integrity, and allows the detection of photosynthetic pigments fluorescence along with morphological observations. The staining protocol has been adjusted for, both, laboratory and natural populations of the genus *Phormidium* (Oscillatoriales), and tested on 4 field-collected samples and 12 laboratory strains of cyanobacteria. Based on the mentioned cellular functions we suggest classification of cells in cyanobacterial populations into four categories: (i) active and intact; (ii) injured but active; (iii) metabolically inactive but intact; (iv) inactive and injured, or dead.

## Introduction

Bacterial populations, including pure cultures in laboratory studies, display high heterogeneity in morphological and physiological activity. It has been widely discussed that individual cells in microbial populations differ substantially in growth rate and in resistance to various stresses [1], [2], [3] that result in a significant cell-to-cell discrepancies in viability and physiological state, becoming more pronounced under stressful conditions. In natural microbial communities this variability is high due to the non-homogeneous physical character of natural environments, irregularity in nutrient distribution and competition between species [4], [5]. Population-based methods, such as respiration measured by the overall oxygen uptake or estimation of photosynthesis performance, provide averaged information on the population's physiological state without considering the properties of single cells, and may result in faulty interpretation of population development and its stress response. Therefore, a versatile approach that estimates multiple physiological parameters at the single-cell level is required for reliable information on the state of the cells in inhomogeneous populations.

The use of fluorochromes for physiological assessment of bacteria provides accurate information about the state of individual cells in populations [6], [7]. A number of fluorescence-based assays that reflect various physiological functions are available for detecting cell viability and activity, such as assessment of membrane integrity and potential, intracellular pH, respiration intensity, intracellular enzymatic activity, etc. [7], [8], [9]. In studies of physiological heterogeneity in populations of microorganisms the fluorochrome staining techniques are often based on detection of only one particular cell function, although multiparameter techniques for bacteria and yeasts have also been established [10], [11], [12], [13]. In cyanobacterial research similar studies, including those where the application of fluorescence dyes are used, are rare and mostly concern unicellular species [14], [15].

The cell is a complex system that responds to a fluctuating environment by modifying its structural organization and by changing its multiple physiological parameters. We consider that a living, healthy and active cyanobacterial cell is primarily characterized by plasma membrane and genome integrities, detectable metabolic activity, and significant content of pigments for effective photosynthetic performance. Under stressful conditions, and due to apoptosis, cells may sustain one or several kinds of damage to their subcellular structures, and changes in their physiological activities.

For the detection and estimation of metabolic activity an assay based on energy dependent processes is required. Respiration is closely bound to the cellular activity [16] and accurately reflects overall energy metabolism of cells. Therefore, detecting respiration is preferable to indirect techniques based on active transport of fluorochromes into the cells, fluorogenic assays for intracellular enzymatic activity, or analysis of photosynthetic performance. Such an estimate may be achieved by employing tetrazolium salts that act as artificial electron acceptors in reaction with the respiratory chain, therefore directly competing with molecular oxygen, and this reaction detects metabolically active cells [17].

The loss of plasma membrane integrity provides a good estimate for bacterial cell viability as it plays a key role in the operation of the whole cell. The maintenance of its integrity is one of the main features discriminating dead or severely injured cells from living cells. Fluorescence assays intended for estimating membrane integrity are based on the passive exclusion of particular dyes (e.g. propidium iodide, SYTOX Green) by cells with structurally integral membranes.

The presence of genetic material is another inherent prerequisite of viability. In cyanobacterial cell, DNA is organized as a compact structure (nucleoid), which is usually located at the center of the cell. The absence of nucleoid or its visibly severe degradation is an obvious feature of non-viable cells. The image of nucleoid morphology may indicate the level of their metabolic activities, since in metabolically inactive cells the irregularly shaped nucleoid may acquire nearly spherical shape due to inhibition of protein synthesis [18]. In addition, the degradation of photosynthetic pigments, either phycobiliproteins or chlorophyll, may also serve as a marker for viability and activity of cyanobacterial cells since it occurs as a response to various stress conditions, and is associated with cell death [15], [19]. In our study we used a cell-permeable dsDNA-specific stain 4′,6-diamidino-2-phenylindole (DAPI) to confirm the presence of nucleoids, and to reveal their shape and localization in SYTOX Green-negative cells.

We present here a novel staining protocol for a multiparameter cell assessment of physiologically heterogeneous populations of cyanobacteria employing three fluorescent dyes combined with microscopy image analysis. Our method allows simultaneous estimation of cellular respiration activity, membrane and genome integrities, and detection of photosynthetic pigment content along with morphology observation. The staining protocol has been adjusted for filamentous species of the genus *Phormidium* (Oscillatoriales). To our knowledge the aforementioned fluorescent probes (and their combinations) have not been previously employed in estimating viability and metabolic activity of filamentous cyanobacteria. Therefore, we tested the efficacy of each fluorescent probe and the specific staining procedure (i.e., incubation time and staining concentration). Simultaneous application of several fluorescent markers required a careful selection of fluorochromes with particular spectral properties, a detailed investigation of their interaction, and their interference with broad-spectrum autofluorescence of the cells (pigments and surface structures). Also, we describe proper handling of the samples and their staining and de-staining procedures due to certain characteristics of the *Phormidium* species, i.e., specific cell-to-cell connections, production of sheaths, and tight structure of natural communities.

## Materials and Methods

### Cyanobacterial material and culture conditions

All laboratory experiments (i.e., concentrations, incubation time, interaction of dyes) were conducted with the *Phormidium autumnale* CCALA 845 strain isolated in 2008 by Snokhousova et Elster from a sample collected in a stream with moss carpets in the Svalbard archipelago (77°00′ 15°20′E), and currently maintained in the Culture Collection of Autotrophic Organisms (CCALA), Institute of Botany, Academy of Sciences of the Czech Republic. Maintaining cultures in liquid or agar BG-11 medium at a continuous photon flux density of 50 µmol·m^–2^·s^–1^ (white light) at +18°C were optimal conditions for the given strain (unpublished data). The incubation time in order to reach the phase of active growth ranged from 15 to 25 days. Because the culture tends to grow in aggregates, and due to the filamentous nature of this strain, estimation of the logarithmical growth phase using optical density of the culture or dry weight increment was not possible. Therefore, the decision regarding the growth phase of the cultures was made on the basis of microscopy observations of the cell morphology (intensive blue-green color, well-pronounced thylakoids, uniform morphology of cells, absence of visible inclusions, absence of necridic cells, and evidence of cell fission). The cultures used for the experiments were at their active growth phase in order to prevent errors in cell counts since “old” cultures are physiologically heterogeneous.

Several strains of cyanobacteria in different stages of growth were also tested with the staining protocol: namely, *Phormidium* species isolated from temperate, tropical and polar environments (CCALA 771, 816, 845, 849, 850, 861, 881), *Calothrix sp.* CCALA 034, *Merismopedia glauca* CCALA 099, *Chroococcidiopsis cubana* CCALA 041, *Oscillatoria limosa* CCALA 134, and *Aphanothece clathrata* CCALA 013.

Besides the examination of laboratory cultures, samples of two natural *Phormidium*-dominated crusts were collected from streaming and stagnant shallow Arctic waters in the Svalbard archipelago in June and September of 2011 and, immediately after collection, used for the fluorescence assays. Both crusts were dominated by *Phormidium autumnale* species identified on the basis of 16S rRNA sequence [20]. We also examined 2 natural samples (mixed epilithic community) collected in the Lužnice River, the Czech Republic in the middle of September 2012. Cyanobacteria in these samples, according to their morphological criteria, were identified as *Phormidium* and *Lyngbya*.

No specific permits were required for the field studies (both in the Czech Republic and in Svalbard). The Svalbard Act of 15 June 2001 No.79 Relating to the Protection of the Environment in Svalbard, states: “The collection of fungi and seaweed for private use is permitted. The collection of flora for research or teaching purposes is permitted where this does not make significant inroads into the local populations of the flora involved.” We confirm that the field studies did not involve endangered or protected species.

### Sample preparation

The laboratory cultures were prepared as follows: cyanobacterial biomass was removed from the growth medium, washed 3 times by shaking and careful draining of liquid with a micropipette, and then resuspended in 300 µL of sterile fresh BG-11 medium in Eppendorf tubes. During the staining procedure the samples were incubated at room temperature in the dark.

The samples of natural crusts were prepared by cutting them into pieces of approximately 1 cm^2^, then thoroughly washed of sand, and transferred to microplates filled with water collected at the sampling sites. The plates were wrapped in aluminum foil, and incubated during the staining procedure at *in situ* outdoor temperature (+2 to +6°C). The soft biomass from the river samples was resuspended in filtered river water, and incubated at +15°C.

### Properties of fluorescent dyes

#### Membrane state

SYTOX Green stain provides several advantages over other dyes used for the same purpose due to its spectral properties and relative non-toxicity to living cells. SYTOX Green is a high affinity nucleic acid stain that does not penetrate living cells, yet it passively diffuses into cells with compromised membranes where it preferentially binds to DNA resulting in >500-fold enhancement in fluorescence emission [21]. The nucleoids of the cells with compromised membranes fluoresce in green color due to the dye uptake. Its bright fluorescence in the green spectral region (absorption and emission maxima at 502 and 523 nm, respectively) allows its observation simultaneously with orange-red fluorescence of CTC (emission maximum at 630 nm), and red autofluorescence of photosynthetic pigments.

#### Respiration

For single-cell evaluation the cell-permeable 5-Cyano-2,3-Ditolyl Tetrazolium Chloride (CTC) and 2-(4-Iodophenyl)-3-(4-nitrophenyl)-5-phenyltetrazolium chloride (INT) redox dyes are the most convenient. These dyes are reduced from soluble colorless form into their corresponding colored (INT) or fluorescent (CTC) insoluble formazans that accumulate intracellularly. The formazan crystals are viewed as intracellular opaque dark-red deposits under transmitted illumination, or as red fluorescent spots (excitation and emission maxima at 488 and 630 nm) when using epifluorescence microscopy. INT was tested as a cheap non-fluorescent analogue of CTC dye; its use may also reduce the cost for an additional microscopy filter cube.

#### Nucleic acids

The DAPI stain was selected for labeling nucleoids due to its brightness and stability; in addition, the probe (Ex/Em maxima at 358/461) is easy to combine with other probes with higher excitation wavelengths. If present, the nucleoids are stained with DAPI in blue (or white-blue) in both living and dead cells [7]. The diffused character of DAPI staining in the nucleoid area, or a whole-cell DAPI signal gives evidence of an extensive damage to genetic material [15].

The properties of the employed fluorescent dyes are summarized in Table 1. The redox dye INT was purchased from Sigma-Aldrich Co. (USA); the redox dye CTC, DNA-binding fluorochrome DAPI, and cell-impermeant nucleic acid dye SYTOX Green were obtained from Life Technologies Corporation (USA). Stock solutions of fluorochromes were prepared and stored according to instructions suggested by the manufacturers.

**Table 1:** Properties of fluorescent dyes.

Fluorochrome	Cell function	Reaction	Detection*
DAPI	Presence/ shape of nucleoids	Stains dsDNA in both living and dead cells	Blue-white (blue) fluorescence of nucleoids
SYTOX Green	Membrane integrity	Stains cells with damaged membranes	Green fluorescence of nucleoids
CTC	Respiratory rate	Accumulation of formazan crystals in active cells	Red-orange fluorescence of CTC-formazan
INT	Respiratory rate	Accumulation of formazan crystals in active cells	Purple deposits under transmitted light

* The colors of the dyes' fluorescence were given by the filter sets that were utilized in our microscopy observations.

### Investigation of the optimal staining procedure of laboratory samples

#### SYTOX Green

To study the minimal SYTOX Green staining concentration, inactivated controls were prepared by immersing the biomass in hot water (10 min at +90°C). They were also prepared by treating the biomass for 60 minutes with 30% (v/v) ethanol water solutions prior to the staining procedure. The samples were then washed free of fixative solution, resuspended in BG-11 medium, and examined within several hours after the fixation procedure. Cell suspensions were stained at final concentrations of 0.05, 0.1, 0.2, 0.3, 0.5, 0.7, 1, 2, and 5 µM of SYTOX Green for 30 min protected from light. The lowest dye concentration that all cells exhibited bright and even fluorescence of their nucleoids was considered as a minimal effective staining concentration.

The maximum staining concentration was optimized by staining non-fixed samples with SYTOX Green solution concentrations of 0.1, 0.2, 0.3, 0.5, 0.7, 1, 1.5, 2, 3, 4, 5, and 10 µM for 30 min in the dark. For each dye concentration, the number of SYTOX Green positive cells and total cell counts were enumerated. At a particular concentration, the SYTOX-positive cell counts were significantly higher compared with the preceding lower concentration of the dye. This lower concentration was considered as maximum.

To find the optimal SYTOX Green incubation times, the samples were stained with a dye concentration (1 µM) in the range between the minimum and maximum staining concentrations. Aliquots of stained suspensions were sampled for investigation after 15, 30, 60, 90, 120, and 150 min of incubation. The minimal staining time of 15 min was set according to the manufacturer's recommendation; the maximum staining time was estimated using a similar technique as for the maximum staining concentration.

#### CTC and INT

When investigating the effect of incubation time on dye reduction we used concentrations of 5 mM CTC and 0.01% w/v INT that was previously employed for direct enumeration of respiring bacteria in environmental and laboratory samples [15], [17]. Subsamples of cell suspensions were collected for microscopy examination after 10 min of staining, and then, in 30 min intervals, during the 2 hour incubation. Cyanobacterial biomass was then fixed with 3% (v/v) formaldehyde water solution and used for slide preparation. The rate of CTC and INT reduction was judged from the quantity and size of red formazan spots in the light field; for CTC the dye red-orange fluorescence in the dark field was also observed. Inactive control was prepared by treating samples with 3% (v/v) formaldehyde water solution for 60 minutes; partially inactivated samples were obtained with subletal formaldehyde concentration of 0.1% (v/v). Additionally, cultures were continuously incubated for 24 hours with CTC or INT dyes to investigate their damaging effect on cells, as previously observed in our preliminary experiments.

The effect of CTC concentrations on dye reduction was conducted with cyanobacterial biomass stained for 30 min in the dark with concentrations of 0.01, 0.05, 0.1, 0.5, 1, 2.5, 5, 7.5, 10 and 20 mM. After incubation, the samples were treated with 3% (v/v) formaldehyde water solution to stop CTC reduction. The quantity, size and pattern of deposition of red formazan spots were observed as well as formazan fluorescence.

#### DAPI

Five µg/mL DAPI concentration, as suggested by the manufacturer, was used in all the experiments. The samples were stained for 15 to 30 min and then rinsed 3 times with BG-11 medium.

### Assessing CTC, DAPI and SYTOX Green interaction, and sequence of staining

Final concentrations of 5 mM CTC, 5 µg/mL DAPI and 1 µM SYTOX Green were used for all the treatments. The effect of SYTOX Green staining on CTC reduction was studied as follows: laboratory samples were dyed with SYTOX Green, after 30 minutes they were washed 3 times, and soaked in fresh BG-11 medium for 15 min to remove excess dye adsorbed on the surfaces of cell walls and sheaths. The biomass was then transferred for staining into a CTC solution for 30 minutes, rinsed in BG-11 medium, and used for microscopy observations. The percentage of respiring cells after SYTOX Green treatment was calculated and the CTC fluorescence pattern was observed; the results of CTC-SYTOX co-staining were compared with the results from samples stained with CTC only. Similarly, to assess the effect of CTC on SYTOX Green counts, 3 sets of samples were prepared: (i) SYTOX Green was used as a single dye; (ii) samples were stained with CTC prior to SYTOX Green; and (iii) SYTOX Green-dyed samples were post stained with CTC. The number of DAPI-positive cells after CTC-SYTOX Green treatment was compared with the cell counts when DAPI alone was used.

### Protocol for CTC, SYTOX Green and DAPI co-staining

Both laboratory and natural samples were stained with SYTOX Green dye in final concentration of 1 µM (10 µM for natural samples) and incubated for 30 min (60 min for natural samples) protected from light. The biomass was then washed 3 to 5 times with sterile BG-11 medium (laboratory samples) or ambient water from the sampling sites (natural samples), and soaked for 15 to 30 min to remove excess dye as necessary. In order to estimate respiratory activity, the samples were post-stained with either INT at final concentrations of 0.01% and 0.03% (w/v), or with 5 mM CTC for 30 min in the dark. After removal of the redox dye solutions, samples were counterstained with DAPI for 15 min (30 min for natural samples) at a final concentration of 5 µg/mL. The samples were then washed thoroughly of the DAPI solution and immersed in fresh media. The same protocol was used for all of the studied laboratory cultures and field samples.

### Microscopy examination and filter spectral properties

To analyze the samples stained with fluorochromes a small aliquot of cyanobacterial suspension was trapped between a glass slide and a 24×24 mm square cover slip; the edges were sealed with wax or nail polish to prevent water evaporation. An Olympus BX53 microscope equipped with a 100 W ultrahigh-pressure mercury arc lamp (Olympus) was used with ×20 and ×40 objectives. The optical system for fluorescence observations included 5 UIS2 fluorescence mirror units (Olympus, Japan): U-FUN filter cube with a 360–370 nm band pass excitation filter and 420 nm long pass cutoff filter for DAPI fluorescence observation; U-FBWA cube with 460–495 nm excitation and 510–550 nm emission filters for SYTOX Green fluorescence, and U-FRFP cube (535–555 nm excitation, 570–625 nm emission ranges) for observing CTC-formazan (CTF) fluorescence. In addition, 3 optional mirror units (excitation filter/emission filter/dichromatic mirror) were made for CTF (425–445 nm/570–625 nm/455 nm), DAPI (360–370 nm/460–510 nm/420 nm), and phycobiliproteins (565–585 nm/600IF/595 nm) fluorescence analyses. The additional mirror units were needed due to the presence of photosynthetic pigments that have broad excitation and emission spectra, mainly in red region. These filters were used in order to avoid overlap between dyes fluorescence (CTC-formazan and DAPI) and pigments autofluorescence. The light absorption and fluorescence emission curves of SYTOX Green and DAPI dyes were redrawn with the permission from www.lifetechnologies.com web source. CTC-formazan graph was taken from the www.olympusmicro.com web source. The formation of INT formazan was observed as optically dense purplish deposits visible with transmitted bright field microscopy.

### Digital image analysis and cell counting

Series of several dark-field images were acquired to record the fluorescence of each of the signals required; and a bright-field image was taken for the total cell counts and detection of INT deposits. The images were captured with an Olympus DP72 microscope digital camera (Japan) at 1024×1360-pixel resolution at a constant shutter, offset and ISO parameters for each filter set. These settings were adjusted to obtain images with the same fluorescence intensity as were seen in microscope eyepieces; a Differential Interference Contrast filter set was used for the bright field image for the enhanced view of the cell septa.

All experiments were run in triplicate on each of the 3 batch cultures grown under the same conditions, i.e., each of the cultures was sampled 3 times during the active growth phase in 2 to 4-day intervals. In experiments investigating concentrations and incubation time, the entire biomass used for staining was observed except the case of SYTOX Green incubation time and SYTOX Green maximum concentration. In two latter and all other experiments, at least 800 randomly chosen cells in 15 to 20 fields of view were counted for each replication.

Image processing was carried out in Adobe Photoshop CS4 program as follows: both bright field image and the corresponding fluorescent image of a microscope view were divided into 20 large squares by applying a grid; 3 to 5 squares were selected for each image, and within these all the cells were counted. Cell counting was performed semi-automatically by using mouse-click counter software (Desktop Counters 2.0, Freelabs); each mouse click marked a counted cell with a dot.

### Statistical Analysis

All statistical analyses of the data were performed in Statistica v.10.0 software (StatSoft, USA). Most of the data did not pass a normality and equal variance test due to uneven distribution of stained cells between microscope fields of view and unequal number of observations per group, which is due to the filamentous nature of the studied strain. Therefore, the Kruskal-Wallis 1-way analysis on ranks at the significance level of 5% was applied to compare the mean of the percentage of stained cells between several groups.

## Results and Discussion

### Application of fluorescent dyes

#### SYTOX Green dye; the effect of staining time and concentration

**Figure 1:**
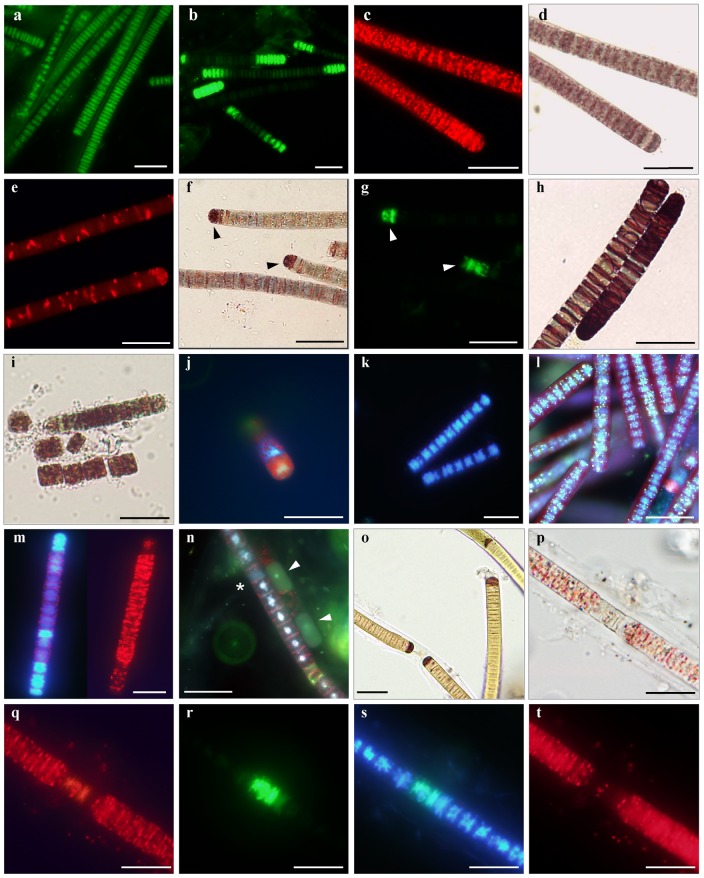
*In situ* detection of cellular functions in cyanobacterium *Phormidium* ** ***autumnale*** **.** **a–l**. Cultures of *Phormidium autumnale* 845 CCALA grown on BG-11 medium. **a.** SYTOX Green staining of formaldehyde-pretreated filaments; **b.** variable staining of live samples after 30 min incubation with 5 µM SYTOX Green; **c–d.** fluorescence and bright field image of CTC-stained filaments in active phase of growth; **e.** partially de-activated cells stained with CTC; **f–g.** INT-treated samples post-stained with SYTOX Green, cells that accumulated INT-formazan (**f**, *arrowheads*) were also SYTOX-positive (**g**, *arrowheads*); **h.** INT-stained filaments in logarithmic phase of growth; **i.** disintegration of filaments after 24-h incubation with CTC; **j.** pattern of CTC-formazan deposition; **k.** DAPI-stained nucleoids in living filaments; **l.** yellow-green metachromatic inclusions in DAPI-stained filaments viewed with a long pass emission filter; **m.** reduced fluorescence intensity of DAPI-stained nucleoids in cells accumulated CTC-formazan (natural samples); **n.** cells from old laboratory cultures simultaneously stained with DAPI, CTC and SYTOX Green under UV-illumination, extensively damaged cells lack SYTOX Green and DAPI staining of nucleoids (*arrowheads*) or have whole-cell DAPI signal (*asterisk*); **o–t.** Field-collected samples of mats in July and September; **o.** accumulation of INT-formazan by the terminal cells of filaments; **p.** a filament with thick sheath stained simultaneously with CTC (**q**), SYTOX Green (**r**), and DAPI (**s**), and showing pigment autofluorescence (**t**). Scale bars are 20 µm.

The minimal effective staining concentration of SYTOX Green dye for laboratory samples of *Phormidium autumnale* was between 0.2 and 0.3 µM in 3 replicates. At this dye concentration all nucleoids in alcohol and heat-pretreated cells exhibited bright and even fluorescence in the green spectral region, which was distinctly visible with a 510–550 optical cutoff filter set (Fig. 1a). Further staining with higher concentrations of the dye (up to 5 µM) did not increase fluorescence intensity. This treatment, as long as it passes through the membranes of the dead cells, confirmed the applicability of SYTOX Green dye to *Phormidium* cultures, since it is also the inactive control.

The mean percentage of SYTOX Green-positive cells in exponentially growing cultures totaled from 0.73% to 1.42%, and was not significantly different at each of the dye concentrations between 0.3 and 3 µM (p>0.05). However, in all treatments, pair wise comparisons revealed a significant rise in percentage of stained cells at concentrations of 4 µM or higher in contrast to each of the preceding concentrations (p<0.05). Thus, the optimal staining concentration for SYTOX Green dye for the 30-minute incubation period for laboratory samples grown under optimal conditions is in the range of 0.3 to 3 µM.

One µM of SYTOX Green dye concentration was selected to approximate the minimal and maximum incubation time required for reliable discrimination between cells with intact vs. compromised membranes. A 15-minute incubation time ensured complete penetration of the dye into the cells with permeabilized membranes. There were no differences (p>0.05) in proportion of SYTOX Green-positive cells between subsamples stained for 15, 30 and 60 minutes; the mean percentage of stained cells increased significantly (p<0.05) in the period between 60 and 90 min. Repeated experiments provided identical results.

Excessive dye concentrations and prolonged incubation periods resulted in heterogeneous staining of cell populations in addition to the increase in proportion of stained cells (Fig. 1b). In these experiments we show that the majority of cells exhibited dim fluorescence of their nucleoids starting at the 3 µM concentration of the dye (30 min incubation) and after 60 min of incubation at 1 µM concentration. At higher concentrations and longer staining periods, the fluorescence increased intensity and varied considerably from cell to cell, which made it difficult to discriminate between stained and non-stained cells. Eventually, at concentration higher than 10 µM the nucleoids of all living cells were brightly stained with SYTOX Green dye.

For the samples of natural population of *Phormidium autumnale*, a SYTOX Green concentration of at least 6 µM was required to label all nucleoids of formaldehyde-pretreated cells; that is 20 times higher than the laboratory samples. At this dye concentration the incubation time sufficient for the penetration of SYTOX Green stain into dead cells exceeded 60 minutes (Fig. 1r). Apparently, this discrepancy may be explained by the tight structure of natural crusts and markedly increased thickness of sheaths enveloping the filaments, which retard the diffusion of the dye into the cells as well as the low (+4°C) ambient temperature. Therefore, we expect that the optimal concentrations of SYTOX Green dye and incubation time may vary significantly from sample to sample depending on the interspecific differences in permeability, state of the cultures, and on conditions the cultures were grown and incubated during the staining procedure. In general, the total labeling of cells in living cultures or inhomogeneous patterns of their staining apparently indicate either excessive dye concentration or inappropriately long incubation time; and accordingly, insufficient dye concentrations or incubation time results in incomplete labeling of cells in permeabilized control samples.

#### Application of CTC and INT dyes

Incubation for 30 min with 0.01, 0.05 and 0.1 mM CTC concentrations were insufficient to invoke the accumulation of any visible formazan deposits, and they were not visible with incubation time that extended for up to 120 min. Among the concentrations analyzed, 0.5 mM of CTC was the lowest concentration that formazan deposits were produced. These deposits were notably smaller than at higher CTC concentrations (starting from 1 mM) that resulted in low fluorescence intensity. The differences in quantity, shape and size of the formazan deposits, as well as the pattern of their deposition, were hardly discernible between 1 and 20 mM.

After 10-min incubation of living laboratory samples with 5 mM concentration of CTC dye, about 99% of the cells in all replicates contained deposits of CTC-formazan visible both by epifluorescence microscopy (Fig. 1c) and under transmitted light (Fig. 1d). Further 2-hour incubation did not lead to any significant increase in the proportion of CTF-containing cells (p>0.05). There was no visible difference between observations in quantity, shape and location of formazan inclusions within the cells for any of the incubation times. However, at incubation periods of 30 min or longer, the CTC-formazan deposits appeared larger, and exhibited brighter fluorescence compared with the deposits produced with 10-min incubation. The same staining protocol was sufficient for penetrating CTC into cells from natural samples even though the filaments were enveloped by thick dense sheaths (Fig. 1p, 1q).

The 1-hour treatment of laboratory *Phormidium* suspensions with 3% (v/v) formaldehyde prior to CTC staining led to a complete suppression of formazan accumulation; this provided a convenient negative control for the absence of any abiotic CTC reduction with the components of the medium used. A short-time pretreatment of samples with subletal formaldehyde concentration (0.1% v/v) resulted in a decrease in the proportion of formazan-containing cells. More important, the CTC-formazan deposits within the cells that retained respiratory activity were markedly different in their numbers, size and shape than from those in non-inactivated cells. Cells in formaldehyde-pretreated samples contained notably fewer sites of CTC reduction per cell, and typically formed larger and more elongated formazan crystals (Fig. 1e) in contrast to the small round formazan spots deposited uniformly throughout the active cells (Fig. 1c). However, the intensity of CTC-formazan fluorescence was not affected in metabolically less active cells. We observed a similar pattern of CTC-formazan deposition within the cells in the old *Phormidium* batch culture (cultivated for 3 to 6 months) and in certain proportion of filaments from field-collected samples collected at the end of the vegetative season on Svalbard (data not shown).

The incubation of the samples with INT revealed that after 10 min only terminal cells had incorporated INT-formazan crystals, whereas other cells within the filaments of neither laboratory nor field-collected samples contain significant formazan deposits (Fig. 1f, 1o). Further 2-hour incubation of laboratory samples resulted in progressive accumulation of INT-formazan from the polar ends across the whole filament. In contrast, subsequent incubation of natural population of *Phormidium* (collected at the end of the vegetative season) for the same amount of time did not lead to any further formazan accumulation, even when the INT concentration was increased to 0.03% (w/v).

Apparently, permeability of the studied cells to INT differs substantially from permeability to CTC. Since the production of formazan crystals is irreversible, the amount of formazan deposited within the cells represents the cumulative respiratory history of those cells [22]. Since the uneven penetration of INT into cells did not reveal differences in their actual respiration rate, we have not observed any visible difference in the amount of formazan accumulated in respiring cells after prolonged incubation of laboratory cultures (>120 min). Evidently, the CTC technique has several other advantages over INT staining when investigating filamentous cyanobacteria. CTC, along with confocal microscopy, may be readily employed in studies of structure and distribution of physiological activity within cyanobacterial communities due to its ability to fluoresce upon reduction. In addition, the production of dense opaque INT-formazan deposits (Fig. 1h) occludes any observations on morphology, intracellular structure, and pigment autofluorescence of cyanobacterial cells as compared with poorly colored and less opaque CTC-formazan (Fig. 1d, 1p).

#### Toxicity of tetrazolium salts

In our studies we discovered that both INT and CTC are toxic to filamentous cyanobacteria, observed from the massive lysis of previously living cells that accumulated visible amounts of formazan during incubation. After an 8-hour continuous incubation with INT and a 24-hour incubation with CTC, the filaments in laboratory cultures disaggregated into single cells (Fig. 1i), and the density of the cultures had dramatically decreased. Similar lysis of cultures occurred, with a delay (after 48 h), when, after a short-time incubation (<60 min), the biomass was washed out of the dye solutions and transferred into a fresh medium. Apparently, the rate of cell lysis positively correlated to the amount of formazan deposited within the cells, and was also connected to a difference in properties of CTC and INT. We also observed that both CTC and INT formazans had well-marked peripheral pattern of deposition (Fig. 1j) within the cells of the filamentous as well as in unicellular cyanobacteria (data not shown).

Tetrazolium salts were frequently reported to be toxic to mixed groundwater and seawater bacterial communities as well as to pure laboratory cultures [17], [23], [24]; however, the mechanism of their toxicity was not explained in these works. As shown for groundwater bacteria [24], the toxicity of CTC and INT dyes was restricted only to those isolates that took up and reduced the dyes into their respective formazans.

As discussed before, the reduction sites of tetrazolium dyes in the electron transport chain of bacteria are strongly species-specific; they are defined by the ability of a particular dye to enter the cells and on the experimental conditions. In studies of *Lactococcus lactis* the electron transport chain components involved in tetrazolium violet reduction included NADH dehydrogenases and menaquinones; in both cases the accumulation of formazan occurred entirely in the cell membranes [25]. The sites of CTC and INT reduction in the aerobic respiratory chain of *E. coli* K-12 were associated with membrane-bound succinate and NADH dehydrogenases. INT was additionally reduced at ubiquinone and possibly cytochromes *b_555, 556_* [26]. The CTC dye was also reduced by the components of plasma membrane ETC rather than by intracellular reductases, and the deposition of the CTC-formazan inflicted damage on plasma membranes [27]. Therefore, it is likely that the toxicity of insoluble tetrazolium formazans may be attributed to a mechanical disruption of the cell membrane produced by the formazan crystals being deposited in close proximity to or within (INT) the inner membrane (CTC and INT). The intramembrane formation of INT-formazan may explain more rapid and severe damage, which INT-formazan created in the studied cells in comparison to CTC-formazan. The fact that formazan-accumulating cells sustain similar damage upon centrifugation (e.g. 10 min at 5000 rpm) may also support the hypothesis of cell injury induced by mechanical disruption of membranes by formazan crystals. However, this does not rule out other mechanisms of spreading their toxicity since water-soluble formazan of XTT terazolium dye (2,3-Bis(2-methoxy-4-nitro-5-sulfophenyl)-2H-tetrazolium-5-carboxanilide inner salt) was also toxic to bacterial isolates upon its reduction [24].

#### SYTOX Green interaction with CTC, INT and DAPI dyes

Since we discovered that deposition of INT and CTC formazans possibly inflicted damage to plasma membranes of studied cyanobacteria, a careful investigation of their effect on staining with membrane-impermeant SYTOX Green dye was required. As we expected, in all replications, the number of SYTOX Green-positive cells in laboratory samples pre-stained with CTC for 30 min was significantly higher than in samples where SYTOX Green was used as a single dye (p<0.05). The proportion of cells labeled with SYTOX Green was 17.5% to 19.3% in CTC-stained samples as compared to 2.18% to 3.24% in non-stained samples. Furthermore, the uneven pattern of SYTOX Green staining within CTC-treated cells was similar to that of cells incubated with excessive concentrations of SYTOX Green dye (Fig. 1b). The samples stained with INT displayed high variability in the SYTOX Green counts. However, most of the cells that incorporated INT-formazan were also distinctly fluorescent with SYTOX Green indicating that an accumulation of INT-formazan did have an effect on the integrity of plasma membranes (Fig. 1f, 1g). For the above mentioned reasons INT dye was not used in any further examination.

In order to employ CTC and SYTOX Green dyes simultaneously, we attempted to stain samples with SYTOX Green, prior to the incubation with CTC, using the same concentrations and staining procedure. The SYTOX Green counts in CTC-treated samples did not differ significantly from the reference samples stained with SYTOX Green only (p>0.05), provided the biomass was thoroughly washed free of the dye solution before CTC staining. Soaking the biomass in fresh medium for 15 to 30 minutes was sufficient to remove residuals of surface-bound SYTOX Green dye. Natural samples required longer time evidently due to the increased density and thickness of their sheaths. Following this procedure some fluorescence of nucleoids was still detectable; however, it was many times less intense when compared to that of non-living control samples. Insufficient washing procedures resulted in populations that were inconsistently stained with SYTOX Green upon further accumulation of CTC-formazan.

There was no effect of SYTOX Green treatment on the ability of studied cells to reduce CTC, i.e., the quantity of formazan crystals and their shape and size were similar to those in the control samples. Despite slightly lower intensity of formazan fluorescence, and the deposits appearing less opaque under transmitted illumination, the fluorescence was still clearly distinguishable from the characteristic formazan deposits produced by partially inactivated cells. In some of the other oscillatorian strains that we tested more CTC-formazan was deposited at the cross walls after SYTOX Green treatment, yet in some strains deposition of CTC-formazan and intensity of its fluorescence were not affected (data not shown).

Five µg/mL concentration of DAPI applied for 15 to 30 min was sufficient to label cell nucleic acids in both laboratory and natural cell populations. Therefore, other concentrations and incubation times were not studied. At 5 µg/mL concentration all observed cells exhibited bright DAPI fluorescence of their nucleoids (Fig. 1k) except for few, apparently, damaged cells. Preceding incubation of samples with SYTOX Green, CTC, INT, or combinations thereof had no effect on DAPI counts, nor did this influence the character of the DAPI staining; yet in some cases we observed interference of the DAPI signal with CTC-fluorescence (see below).

#### Protocol for co-staining of laboratory and natural samples

*Phormidium* species isolated from temperate, tropical and polar environments (CCALA 771, 816, 845, 849, 850, 861, 881), also *Calothrix sp.* CCALA 034, *Merismopedia glauca* CCALA 099, *Chroococcidiopsis cubana* CCALA 041, *Oscillatoria limosa* CCALA 134, *Aphanothece clathrata* CCALA 013, at different stages of growth were successfully stained using the protocol developed for the laboratory samples. The efficiency of the staining protocol was evaluated according to the following criteria: (i) all cells in inactivated controls were brightly stained with SYTOX Green and lacked CTC-formazan deposits; (ii) all cells were stained with DAPI except the apparently damaged cells; (iii) SYTOX-positive cells in living samples were, in general, clearly distinguishable from SYTOX-negative cells; (iv) CTF-containing cells were not selectively stained with SYTOX Green, or the number of SYTOX-positive cells did not increase after staining with CTC; (v) filaments (Oscillatoriales) were not disaggregated into single cells or short fragments after staining procedure; (vi) SYTOX Green treatment prior to CTC did not suppress formazan reduction; (vii) most of the cells in actively growing controls were not stained with SYTOX Green and accumulated formazan deposits. Using this approach, the staining protocol established for actively growing laboratory cultures was also valid for the same cultures at later phases of growth (data not shown).

Although the developed protocols were suitable for all studied cultures, we expect that different organisms may require different staining protocols. If studied the culture/sample does not meet the aforementioned criteria, then staining concentrations, incubation time and/or different washing procedure should be tested. The reported optimal concentrations and incubation times may vary substantially depending on the nature of the samples [14], [15], [17], [21], [23], [24], [28], [29]. In initial experiments, it is worth to try several dye concentrations and incubation times to determine those that yield optimal staining. Following the above mentioned criteria, we performed optimization experiments for natural samples. The staining protocol was primarily developed for one of the field-collected Svalbard samples, and was suitable for all 4 studied natural populations of cyanobacteria.

### Spectral interaction of fluorescent probes

The number of fluorescent dyes that may be simultaneously introduced into a specimen is generally limited by the relatively narrow spectrum of visible light. Therefore, a careful selection of fluorescent probes and microscopy filters is required so no overlap in their fluorescence signals (including pigment and cell surface autofluorescence) exists. This will provide reliable criteria for their discrimination if interference between signals cannot be avoided.

#### Autofluorescence of photosynthetic pigments

Phycobiliproteins, as accessory light-harvesting pigments of cyanobacteria, have wide absorption and emission spectra that significantly restricts employing other fluorochromes. The pigments typical for Oscillatoriales allophycocyanin (APC), C-phycocyanin (C-PC), and optionally C-phycoerythrin (C-PE), absorb light between 500 and 700 nm [30], [31], [32], [33]. The tight energetic coupling between phycobiliproteins of the phycobilisome [31], results in almost complete lack of fluorescence from C-PC and C-PE [31], [34], [35]. The emission of intact phycobilisomes arises mainly from far-red-emitting APC forms with a peak between 670 and 675 nm [34] (Fig. S1b) along with red fluorescence of chlorophyll *a,* mostly in the 675–695 nm range [32], [36], [37].

Light in the 565–585 nm range is sufficient to excite both C-PE and C-PC, and to some extent APC (Fig. S1a) and corresponds to the absorption peak of intact phycobilisomes from *Phormidium percisinum* grown under white light [38]. The composition of phycobiliproteins is dependent on the quality and the intensity of light as well as on nutrient conditions [33]; therefore, longer wavelength excitation may be useful for cultures containing predominantly APC pigments.

#### CTC fluorescence

The selected 425–445 nm excitation range corresponds to the side absorption peak of CTC-formazan, and ensures its exclusive excitation since the absorption spectra of phycobiliproteins, SYTOX Green, and DAPI are not included in this region (Fig. S2a, S2b). The red fluorescence of chlorophyll *a* (excitation peak at 425 nm) was filtered out with a 625 nm cutoff filter. The fluorescence of formazan crystals was then readily observed against the low background noise given by the properties of the filter set used. The 530–550 nm excitation range was suitable for the observation of natural samples due to the lack of background noise and brighter fluorescence of CTC-formazan; but was not applicable to laboratory cultures apparently due to the difference in the composition of the phycobiliproteins.

#### SYTOX Green and DAPI fluorescence

Green fluorescence from SYTOX Green-stained nucleoids was selectively observed with a 510–550 nm band pass barrier filter as the only signal available under given 460–495 nm excitation range (Fig. S2a).

Long-wave UV light (360–370 nm) required for the maximum excitation of dsDNA-bound DAPI (Fig. S2b) invoked concurrent excitation of CTC-formazan and SYTOX Green dye as well as photosynthetic pigments. A 420IF barrier filter was inappropriate for the observation of exponentially growing cells due to the shielding of the DAPI signal by intense pigment autofluorescence. However, it was useful for the simultaneous observation of CTC-formazan, DAPI, and SYTOX Green fluorescence in cells from old cultures (incubated for 3 to 6 months) with low photosynthetic pigment content (Fig. 1n). The intensity of DAPI fluorescence was low with a 420–460 nm barrier filter even though the emission maximum of DAPI (461 nm) extended into this range. The 460–510 nm barrier filter ensured bright fluorescence of DAPI-labeled nucleoids (Fig. 1k), and sufficiently eliminated signals of the other fluorochromes, including green-yellow fluorescence of DAPI-accumulating metachromatic inclusions (Fig. 1l), apparently intracellular polyphosphates [7].

#### Overlaps in fluorescence signals

The optimal combination of filter sets allowed for individual detection of fluorescence signals from all the fluorochromes employed. Nevertheless, in certain cases we observed an overlap in dye emission spectra, and their interference with autofluorescence of cell components. We detected autofluorescence of the entire filament surface with both 510–550 nm and 575–625 nm emission filters when samples were excited with blue or blue-violet light, yet the fluorescence signals from SYTOX Green-stained nucleoids and CTC-formazan crystals due to their higher intensity and their characteristic staining pattern contrasted vividly with the autofluorescence background. The fluorescence of SYTOX Green-labeled nucleoids of dead cells is visible with 425–445 nm/575–625 nm filter set, although the SYTOX Green dye absorbs light of this spectrum poorly (Fig. S2a). This overlap did not hinder the detection of the CTC-formazan signal because of the lack of respiratory activity in severely injured or dead cells. Although the data obtained from www.olympusmicro.com indicates that CTC-formazan is not excited by light longer than cca. 525 nm, it was reported that light up to 560 nm was optimal to excite CTC-formazan incorporated into bacterial cells [23], [24]. Thus we observed the red fluorescence of CTC-formazan with 565–585 nm/600IR nm filter set, provided the cells lacked photosynthetic pigments absorbing light in this range (Fig. 1t). In addition, the presence of CTC-formazan within the cells suppressed the DAPI fluorescence in some of the filaments in the natural samples. The fluorescence of such DAPI-labeled nucleoids was notably lower, yet still detectable (Fig. 1m). It seems that the structures within the cells stained with DAPI may come, at some point, into close spatial proximity of CTC-formazan deposits resulting in dye-to-dye interaction, which we observed in some of the natural samples. This interaction may be explained by the absorption of photons, emitted by DAPI, or by Forster energy transfer due to the overlap of the DAPI emission with the CTC-formazan absorption [29].

### Sample handling during staining procedure and microscopy observation

The filamentous architecture of oscillatorian cyanobacteria and their tight cell-to-cell connections require certain precautions during the staining procedure and preparation of samples. We recommend avoiding extensive breakage of colonies into fine suspension, and long centrifugation at high speed since the cells usually sustain damage at the site of the filament disruption. We observed that after such treatment the polar cells of the filaments turn SYTOX Green-positive, and accumulate higher amounts of CTC-formazan, apparently due to injuries to their plasma membranes.

Dispersal of tight *Phormidium* colonies is required to permit the dyes a direct contact with the cells. Because the cells, which incorporate CTC-formazan, become increasingly sensitive to any mechanical manipulations, removal of staining solutions should be done through careful draining of the liquid with a micropipette rather than centrifugation. If the staining solution cannot be separated in this manner it is possible to use gentle centrifugation (e.g. 1-2 min at 1000 rpm) or cell filtration.

The placement of an organism into an environment different in composition from the growth medium may result in an increase or a decrease in its respiratory activity. Therefore, as a solution for staining and washing we used complete or diluted BG-11 medium and incubated the samples under the same conditions as those used for cultivation. We also stress that many experiments require careful consideration of conditions for incubation in the context of aims and desirable results. This is especially true, for example, when the effect of osmotic, freezing, or nutrient stress is being studied. Once the staining procedure is completed, a different solution can be used for microscopy observation to eliminate background fluorescence. It is also worth noting that cyanobacterial cells, usually larger than other bacteria, may easily be destroyed during the preparation of microscopy slides, necessitating the spreading of the biomass onto the slide carefully, and sealing the edges of the cover slips to prevent an increase of tension between the glass surfaces as the water evaporates.

The damaging effect of excitation light on fluorochromes should also be considered in microscopy observations. To minimize the extent of pigment photobleaching and light-induced degradation of fluorescent probes, the excitation light of longer wavelengths is recommended to be applied first. The observation of phycobiliproteins, which absorbs light in the long-wave region, should be followed by SYTOX Green (blue excitation), CTC-formazan (blue-violet), and DAPI (UV) fluorescence observations. The bleaching effect of short-wave (UV) light through de-coloration of CTC-formazan may be advantageous in the case when the investigation of cell morphology and intracellular structure is required. Alternatively, an optical system based on light-emitting diodes (LED) may be employed for fluorescence microscopy observations in order to provide higher contrast and to reduce damages produced by mercury lamp irradiation, i.e., phototoxicity and heat effect on a specimen, and photobleaching of the fluorescent dyes. LED light sources with a flexible modular design (e.g., Colibri LED light source system from Carl Zeiss MicroImaging) allow rapid changes in the excitation light of the required wavelength, making this system suitable for multicolor fluorescence microscopy.

### Interpretation of data and limitations of the method

As discussed, the cell physiology and activity do not appear as discrete variables – they are rather represented as a continuum of various physiological processes [5]. Apparently, there is a variety of states between obviously ‘dead’ and ‘living’ cells in microbial assemblages. Such cell states may display reduced overall metabolic activity or its particular processes, and the structural and functional integrity of their cellular compounds may be different. For this reason, the cells in the populations must be grouped into broad artificial categories depending on the operational definitions given for cell activity and viability. It should be mentioned that no single viability test provide reliable information of the cell state, and the classification of the cells into particular groups is usually determined by the combination and sensitivity of selected assays.

Although the criteria for cell death and viability have been broadly debated [1], the absence of genetic material in the nucleoid area or its obvious disintegration provides a clear evidence of cell non-viability [39]. However, the degradation of DNA may occur with a delay after the cessation of physiological processes and the loss of membrane integrity, which prevents certification of viability of nucleoid-containing cells [39], [40].

The use of ‘dead cell’ stains (SYTOX Green) that reveal the structural disintegration of plasma membranes, is often considered useful for detecting non-viable cells, yet a positive reaction to such staining may indicate cell injury that can be subsequently followed by recovery or by death of the cell. In certain cases, algal communities do not show bimodal distribution of SYTOX Green-positive and SYTOX Green-negative cells. Instead, they show a more complex staining feature that is displayed as variability of fluorescence intensity from SYTOX Green-stained nucleoids [14]. Nevertheless, the SYTOX Green dye may be employed to discriminate between live, injured and dead cells by selecting different thresholds of intensity of the fluorescence signal. Respiration rate measurement, in general, may provide a good estimate for cell activity; it also indicates cell viability to some extent as respiration is attributed only to live cells. However, this assay should be interpreted in conjunction with other cellular functions since injured cells can still maintain significant rates of respiration.

The amount of formazan incorporated into cells does not necessarily correspond to the actual respiratory activity, e.g., intensive and rapid CTC reduction may reflect the increased permeability of the membrane barrier of injured cells, unlike permeability of intact cells that display slower accumulation of CTC-formazan [23].

The lack of phycobiliproteins or changes in their composition is influenced by environmental conditions, and may be attributed to cells in different physiological states. A living and active cell is expected to have significant amounts of phycobiliproteins. Indeed, the high content of accessory photosynthetic pigments may indicate an abundance of nitrogen sources in the environment that accumulate to form phycobiliprotein aggregates. Availability and composition of phycobiliproteins are also determined by the intensity of light or by its spectral properties. It was shown that non-viable cyanobacterial cells, after a long-term desiccation, undergo pigment bleaching [15]; their rapid decay upon cell death was also observed in marine phytoplankton [14]. However, phycobiliproteins can still be present in dead, injured, or metabolically inactive cells. In this case, their autofluorescence is detectable, but the output is possibly lower and of different wavelengths; e.g., stress and unfavorable conditions provoke the dissociation of phycobilisome subunits [34], [35]. This results in increased fluorescence emission from C-PE and C-PC, and a drop in chlorophyll autofluorescence. To summarize, the presence and intensity of pigment fluorescence may provide additional information regarding the state of the cells and their stress response, although it cannot serve, by itself, as a robust marker of activity level and viability of cyanobacterial cells. Accurate and detailed data regarding the physiology of the photosynthetic apparatus and its connection to the cell state may be obtained by employing chlorophyll fluorescence analysis [41], [42], which, should not be used in combination with fluorescent probes because they generally have negative effects on the cell physiology and may interfere with the chlorophyll fluorescence signal.

**Figure 2:**
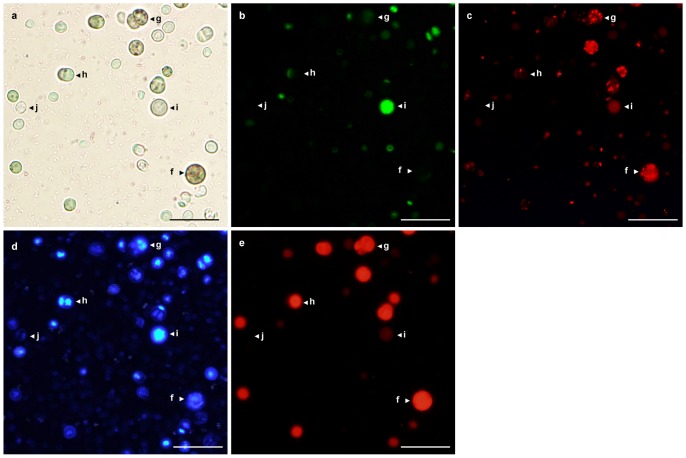
Living 3-month-old *Chroococcidiopsis* 041 CCALA laboratory culture (a) simultaneously stained with SYTOX Green (b), CTC (c) and DAPI (d) dyes, and showing pigment autofluorescence (e).

We propose that cells in cyanobacterial populations can be conventionally classified into several categories: (i) active and intact; (ii) injured but active; (iii) metabolically inactive but intact; (iv) inactive and injured, or dead. Through the use of a set of physiological markers employed by this method (respiratory rate, membrane integrity, the presence and integrity of a nucleoid, photosynthetic pigments), it is possible to distribute the cells among the above mentioned groups (Table 2, Fig. 2). Criteria for classifications of cells in heterogeneous bacterial populations have also been previously suggested in the papers by Caron and Badley, 1995 [43], Kell et al., 1998 [1] and Del Giorgio and Gasol, 2008 [5].

**Table 2:** Classification of cells according to the selected criteria.

Physiological state of a cell	CTC reduction	SYTOX Green positive	DAPI positive	Photosynthetic pigments	Fig. 2, marked as
Active and intact (living healthy cell)	+	−	+	+	f
Injured but active (living injured or apoptic cell)	+	+	+	+/−	g
Metabolically inactive but intact (presumably dormant cell)	−	−	+	±/−	h
Inactive and injured (nucleoid-containing dead cells)	−	+	+	±/−	i
Non-nucleoid-containing dead cells	−	−	−	−	j

## Conclusion

The method introduced is useful for studies of cyanobacterial population development and stress response. Our method allows rapid multiparameter assessment of the state of individual cells that may display substantial multimodal variability in both natural and laboratory populations. Although it provides a simplified view of physiological heterogeneity in populations of cyanobacteria (since it covers only limited spectrum of cell functions), our method allows a quick estimate on the condition of a large number of individual cells, and discriminate living, injured, dormant and dead cells according to the suggested criteria. Other than those described here, investigations of single-cell physiology include a number of advanced techniques summarized in reviews by Lidstrom and Konopka, 2010 [3], and Müller and Caron, 2010 [7].

## Supporting Information

Figure S1 **Absorption (a) and emission (b) spectra of C-phycoerythrin (grey), C-phycocyanin (red), and allophycocyanin (blue).** The excitation and emission ranges of microscopy filters are denoted as spaces between dotted lines. The graphs were modified from Mimuro et al. [32], Ying and Xie [44], and Teale and Dale [30].
(TIF)

Figure S2 **Spectral profiles of the fluorescent probes used.** The curves represent absorption and emission properties of SYTOX Green (**a**) and DAPI (**b**). The excitation (left chart) and emission (right chart) ranges of microscope filters are denoted as spaces between dotted lines. The light absorption and fluorescence emission curves of SYTOX Green and DAPI dyes were redrawn with the permission from www.lifetechnologies.com web source.
(TIF)

## References

[1] Kell DB, Kaprelyants AS, Weichart DH, Harwood CR, Barer MR (1998) Viability and activity in readily culturable bacteria: a review and discussion of the practical issues. Antonie Van Leeuwenhoek 73(2): 169–187. 9717575 10.1023/a:1000664013047

[2] Kell DB, Young M (2000) Bacterial dormancy and culturability: the role of autocrine growth factors. Curr Opin Microbiol 3(3): 238–243. 10851153 10.1016/s1369-5274(00)00082-5

[3] Lidstrom ME, Konopka MC (2010) The role of physiological heterogeneity in microbial population behavior. Nat Chem Biol 6(10): 705–712. 20852608 10.1038/nchembio.436

[4] Davey HM, Winson MK (2003) Using flow cytometry to quantify microbial heterogeneity. Curr Issues Mol Biol 5(1): 9–15. 12638660

[5] Del Giorgio PA, Gasol GM (2008) Physiological structure and single-cell activity in marine bacterioplankton. In: Microbial ecology of the oceans, Second Edition, Kirchman DL, editor. John Wiley & Sons, Inc., Hoboken, NJ, USA, 243–298.

[6] Davey HM, Kell DB (1996) Flow cytometry and cell sorting of heterogeneous microbial populations: the importance of single-cell analyses. Microbiol Rev 60(4): 641–696. 8987359 10.1128/mr.60.4.641-696.1996 PMC239459

[7] Müller S, Caron NG (2010) Functional single-cell analyses: flow cytometry and cell sorting of microbial populations and communities. FEMS Microbiol Rev 34(4): 554–587. 20337722 10.1111/j.1574-6976.2010.00214.x

[8] McFeters GA, Yu FP, Pyle BH, Stewart PS (1995) Physiological assessment of bacteria using fluorochromes. J Microbiol Methods 21(1): 1–13. 11538412 10.1016/0167-7012(94)00027-5

[9] Breeuwer P, Abee T (2000) Assessment of viability of microorganisms employing fluorescence techniques. Int J Food Microbiol 55(1–3): 193–200. 10791743 10.1016/s0168-1605(00)00163-x

[10] Porter J, Edwards C, Pickup RW (1995) Rapid assessment of physiological status in Escherichia coli using fluorescent probes. J Appl Bacteriol 79(4): 399–408. 7592133 10.1111/j.1365-2672.1995.tb03154.x

[11] López-Amorós R, Castel S, Comas-Riu J, Vives-Rego J (1997) Assessment of E. coli and Salmonella viability and starvation by confocal laser microscopy and flow cytometry using rhodamine 123, DiBAC4(3), propidium iodide, and CTC. Cytometry 29(4): 298–305. 9415412 10.1002/(sici)1097-0320(19971201)29:4<298::aid-cyto6>3.0.co;2-6

[12] Caron NG, Stephens P, Badley R (1998) Assessment of bacterial viability status by flow cytometry and single cell sorting. J Appl Microbiol 84(6): 988–998. 9717283 10.1046/j.1365-2672.1998.00436.x

[13] Hernlem B, Hua SS (2010) Dual fluorochrome flow cytometric assessment of yeast viability. Curr Microbiol 61(1): 57–63. 20049598 10.1007/s00284-009-9576-7

[14] Veldhuis M, Kraay G, Timmermans K (2001) Cell death in phytoplankton: correlation between changes in membrane permeability, photosynthetic activity, pigmentation and growth. Eur J Phycol 36(2): 167–177.

[15] Billi D (2009) Subcellular integrities in Chroococcidiopsis sp. CCMEE 029 survivors after prolonged desiccation revealed by molecular probes and genome stability assays. Extremophiles 13(1): 49–57. 18931823 10.1007/s00792-008-0196-0

[16] Zimmermann R, Iturriaga R, Becker-Birck J (1978) Simultaneous determination of the total number of aquatic bacteria and the number thereof involved in respiration. Appl Environ Microbiol 36(6): 926–935. 367268 10.1128/aem.36.6.926-935.1978 PMC243168

[17] Rodriguez GC, Phipps D, Ishiguro K, Ridgway HF (1992) Use of a fluorescent redox probe for direct visualization of actively respiring bacteria. Appl Environ Microbiol 58: 1801–1808. 1622256 10.1128/aem.58.6.1801-1808.1992 PMC195687

[18] Robinow C, Kellenberger E (1994) The bacterial nucleoid revisited. Microbiol Rev 58: 211–232. 7521510 10.1128/mr.58.2.211-232.1994 PMC372962

[19] Schwarz R, Forchhammer K (2005) Acclimation of unicellular cyanobacteria to macronutrient deficiency: emergence of a complex network of cellular responses. Microbiology 151(8): 2503–2514. 16079330 10.1099/mic.0.27883-0

[20] Strunecký O, Komárek J, Elster J (2012) Biogeography of Phormidium autumnale (Oscillatoriales, Cyanobacteria) in western and central parts of Spitsbergen. Polish Polar Research: In press.

[21] Roth BL, Poot M, Yue ST, Millard PJ (1997) Bacterial viability and antibiotic susceptibility testing with SYTOX green nucleic acid stain. Appl Environ Microbiol 63: 2421–2431. 9172364 10.1128/aem.63.6.2421-2431.1997 PMC168536

[22] Schaule G, Flemming H-C, Ridgway HF (1993) Use of 5-cyano-2,3-ditolyl tetrazolium chloride for quantifying planktonic and sessile respiring bacteria in drinking water. Appl Environ Microbiol 59: 3850–3857. 8285688 10.1128/aem.59.11.3850-3857.1993 PMC182540

[23] Kaprelyants AS, Kell DB (1993) The use of 5-cyano-2,3-ditolyl tetrazolium chloride and flow cytometry for visualization of respiratory activity in individual cells of Micrococcus luteus. J. Microbiol. Methods 17: 115–122.

[24] Hatzinger PB, Palmer P, Smith RL, Peñarrieta CT, Yoshinari T (2003) Applicability of tetrazolium salts for the measurement of respiratory activity and viability of groundwater bacteria. J Microbiol Methods 2(1): 47–58. 10.1016/s0167-7012(02)00132-x 12401226

[25] Tachon S, Michelon D, Chambellon E, Cantonnet M, Mezange C, et al (2009) Experimental conditions affect the site of tetrazolium violet reduction in the electron transport chain of Lactococcus lactis. Microbiology 155: 2941–2948. 19520722 10.1099/mic.0.029678-0

[26] Smith JJ, McFeters GA (1997) Mechanisms of INT (2-(4-iodophenyl)3-(4-nitrophenyl)-5-phenyl tetrazolium chloride) and CTC (5-cyano-2,3-ditolyl tetrazolium chloride) reduction in Escherichia coli K-12. J Microbiol Methods 29: 161–175. 10.1111/j.1365-2672.1996.tb03212.x 8642015

[27] Bernas T, Dobrucki JW (2000) The role of plasma membrane in bioreduction of two tetrazolium salts, MTT, and CTC. Arch Biochem Biophys 380(1): 108–16. 10900139 10.1006/abbi.2000.1907

[28] Schumann R, Schiewer U, Karsten U, Rieling T (2003) Viability of bacteria from different aquatic habitats. II. Cellular fluorescent markers for membrane integrity and metabolic activity. Aquatic Microbial Ecology 32: 137–150.

[29] Yu W, Dodds W, Banks M (1995) Optimal staining and sample storage time for direct microscopic enumeration of total and active bacteria in soil with two fluorescent dyes. Appl Environ Microbiol 61(9): 3367–3372. 16535124 10.1128/aem.61.9.3367-3372.1995 PMC1388578

[30] Teale FW, Dale RE (1970) Isolation and spectral characterization of phycobiliproteins. Biochem J 116(2): 161–169. 5414092 10.1042/bj1160161 PMC1185341

[31] Bryant DA (1982) Phycoerythrocyanin and phycoerythrin: properties and occurrence in cyanobacteria. J Gen Microbiol 128(4): 835–844.

[32] Mimuro M, Kikuchi H, Murakami A (1999) Structure and function of phycobilisomes. In: Concepts in photobiology: photosynthesis and photomorphogenesis. Singhal GS, Regner G, Sopory SK, Irrgang K-D, editors. Boston: Kluwer Academic Publishers; Delhi: Narosa Pub House. 104–135.

[33] Bodemer U (2004) Variability of phycobiliproteins in cyanobacteria detected by delayed fluorescence excitation spectroscopy and its relevance for determination of phytoplankton composition of natural water samples. J Plankton Res 26(10): 1147–1162.

[34] Gantt E, Lipschultz CA, Grabowski J, Zimmerman BK (1979) Phycobilisomes from blue-green and red algae: isolation criteria and dissociation characteristics. Plant Physiol 63: 615–620. 16660778 10.1104/pp.63.4.615 PMC542883

[35] Rigbi M, Rosinski J, Siegelman HW, Sutherland JC (1980) Cyanobacterial phycobilisomes: Selective dissociation monitored by fluorescence and circular dichroism. Proc Natl Acad Sci U S A 77(4): 1961–1965. 16592802 10.1073/pnas.77.4.1961 PMC348629

[36] French CS, Smith JHC, Virgin HI, Airth RL (1956) Fluorescence-spectrum curves of chlorophylls, pheophytins, phycoerythrins, phycocyanins and hypericin. Plant Physiol 31: 369–374. 16654902 10.1104/pp.31.5.369 PMC540806

[37] Brown JS (1969) Absorption and fluorescence of chlorophyll a in particle fractions from different plants. Biophys J 9(12): 1542–1552. 5352230 10.1016/S0006-3495(69)86469-6 PMC1367651

[38] Siegelman HW, Kycia JH (1982) Molecular morphology of cyanobacterial phycobilisomes. Plant Physiol 70(3): 887–897. 16662595 10.1104/pp.70.3.887 PMC1065790

[39] Zweifel UL, Hagstrom A (1995) Total counts of marine bacteria include a large fraction of non-nucleoid-containing bacteria (ghosts). Appl Environ Microbiol 61(6): 2180–2185. 16535043 10.1128/aem.61.6.2180-2185.1995 PMC1388461

[40] Karner M, Fuhrman J (1997) Determination of active marine bacterioplankton: a comparison of universal 16S rRNA probes, autoradiography, and nucleoid staining. Appl Environ Microbiol 63(4): 1208–1213. 16535563 10.1128/aem.63.4.1208-1213.1997 PMC1389541

[41] Campbell D, Hurry V, Clarke AK, Gustafsson P, Oquist G (1998) Chlorophyll fluorescence analysis of cyanobacterial photosynthesis and acclimation. Microbiol Mol Biol Rev 62(3): 667. 9729605 10.1128/mmbr.62.3.667-683.1998 PMC98930

[42] Maxwell K, Johnson GN (2000) Chlorophyll fluorescence – a practical guide. J Exp Bot 51(345): 659–668. 10938857 10.1093/jxb/51.345.659

[43] Caron NG, Badley RA (1995) Viability assessment of bacteria in mixed populations using flow cytometry. J Microsc 179(1): 55–66.

[44] Ying L, Xie XS (1998) Fluorescence spectroscopy, exciton dynamics, and photochemistry of single allophycocyanin trimers. J Phys Chem B 102(50): 10399–10409.

