# Supplementary figures and images for: A Novel Staining Protocol for Multiparameter Assessment of Cell Heterogeneity in Phormidium Populations (Cyanobacteria) Employing Fluorescent Dyes
Source: PLoS One. 2013 Feb 20;8(2):e55283. doi: 10.1371/journal.pone.0055283 (PMC3577823; doi:10.1371/journal.pone.0055283)

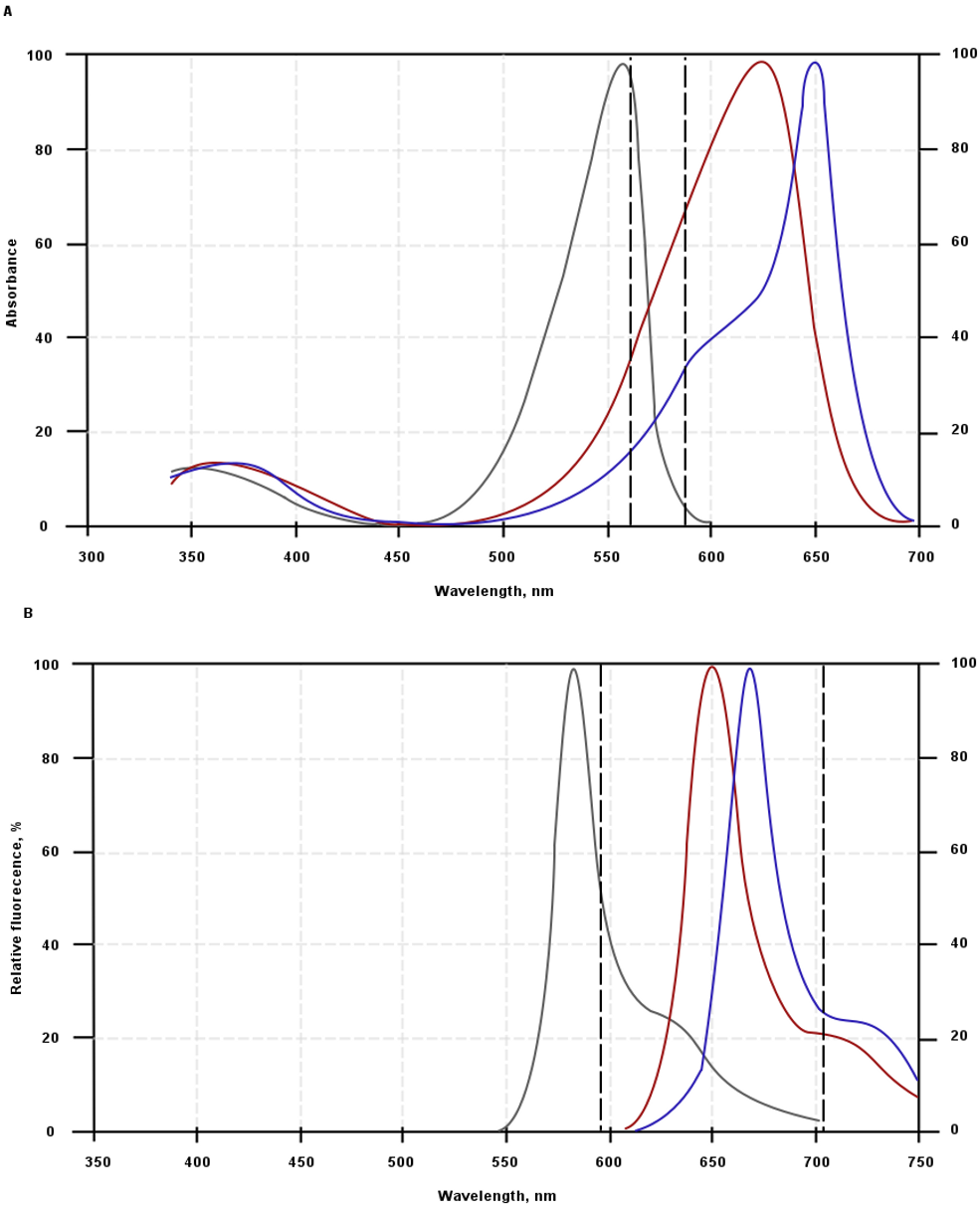

Supplement: Figure S1 — Absorption (a) and emission (b) spectra of C-phycoerythrin (grey), C-phycocyanin (red), and allophycocyanin (blue). The excitation and emission ranges of microscopy filters are denoted as spaces between dotted lines. The graphs were modified from Mimuro et al. [32], Ying and Xie [44], and Teale and Dale [30]. (TIF) [file pone.0055283.s001.tif]

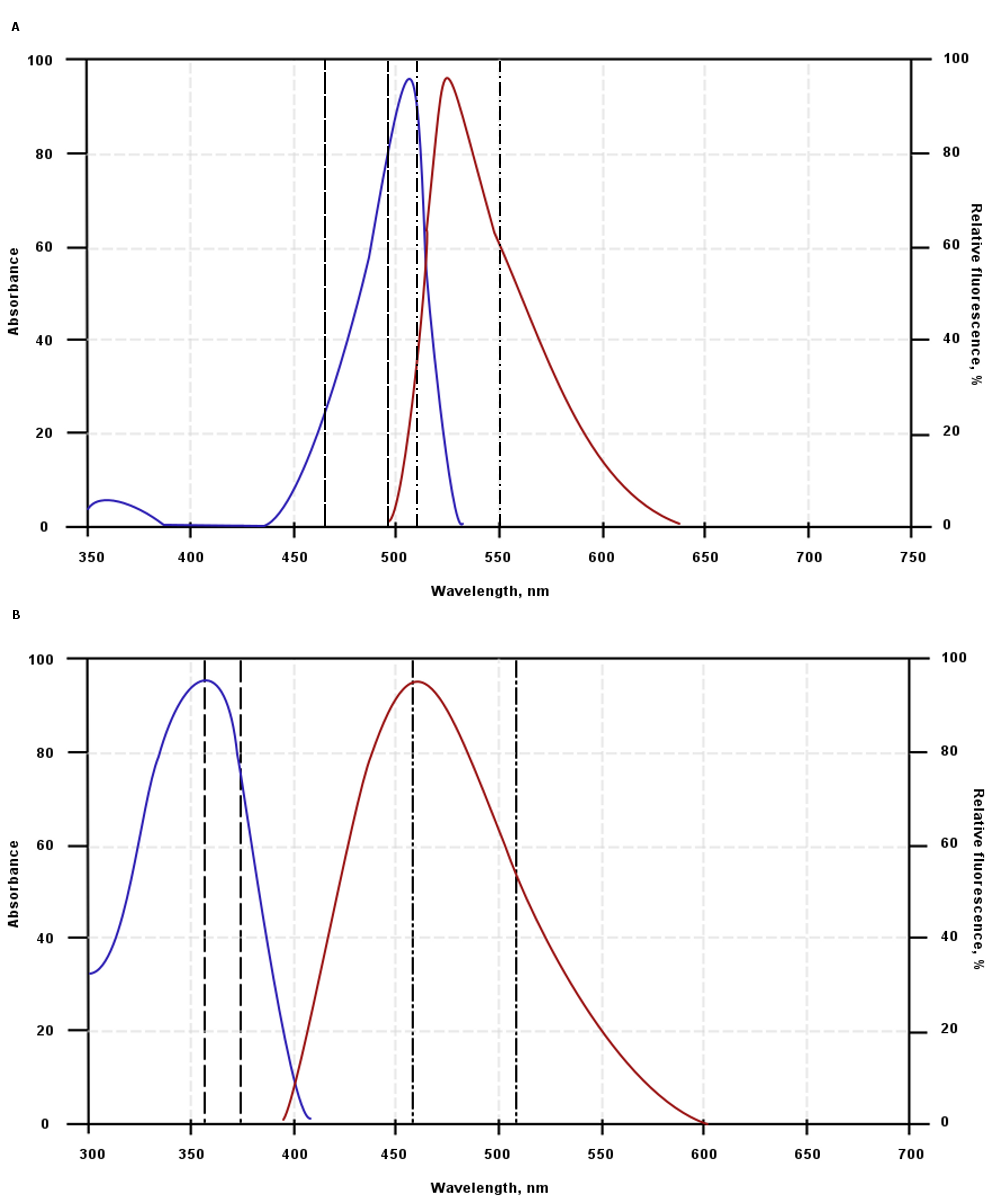

Supplement: Figure S2 — Spectral profiles of the fluorescent probes used. The curves represent absorption and emission properties of SYTOX Green (a) and DAPI (b). The excitation (left chart) and emission (right chart) ranges of microscope filters are denoted as spaces between dotted lines. The light absorption and fluorescence emission curves of SYTOX Green and DAPI dyes were redrawn with the permission from www.lifetechnologies.com web source. (TIF) [file pone.0055283.s002.tif]
